# The Influence of Expectation on Nondeceptive Placebo and Nocebo Effects

**DOI:** 10.1155/2018/8459429

**Published:** 2018-03-19

**Authors:** Hua Wei, Lili Zhou, Huijuan Zhang, Jie Chen, Xuejing Lu, Li Hu

**Affiliations:** ^1^CAS Key Laboratory of Mental Health, Institute of Psychology, Beijing, China; ^2^Department of Psychology, University of Chinese Academy of Sciences, Beijing, China; ^3^Faculty of Psychology, Southwest University, Chongqing, China; ^4^Cognition and Human Behavior Key Laboratory of Hunan Province, Hunan Normal University, Changsha, Hunan, China

## Abstract

Nondeceptive placebo has demonstrated its efficiency in clinical practice. Although the underlying mechanisms are still unclear, nondeceptive placebo effect and nondeceptive nocebo effect may be mediated by expectation. To examine the extent to which expectation influences these effects, the present study compared nondeceptive placebo and nocebo effects with different expectation levels. Seventy-two healthy female participants underwent a standard conditioning procedure to establish placebo and nocebo effects. Sequentially, participants were randomized to one of the four experimental groups—baseline (BL), no expectation intervention (NoEI), expectation increasing (EI), and expectation decreasing (ED) groups, to receive either no intervention or interventions through different verbal suggestions that modulated their expectation. Placebo and nocebo effects were established in all four groups after the conditioning phase. However, after disclosing the placebo and nocebo, the analgesic and the hyperalgesic effects only persisted in the EI group, when compared with the BL group. Our results provide evidence highlighting the critical role of increased expectation in nondeceptive placebo and nocebo effects. The finding suggests that open-label placebo or nocebo per se might be insufficient to induce strong analgesic or hyperalgesic response and sheds insights into administrating open-label placebo and avoiding open-label nocebo in clinical practice.

## 1. Introduction

Placebo effect is a psychobiological effect that occurs following the administration of a placebo, that is, an inert treatment [1]. In most studies, a sham substance or sham equipment was administrated deceptively to induce individual's expectation of placebo, promoting the treatments for pain, motor disorders, anxiety, depression, and other diseases [1–6]. Given that disclosing the placebo may reduce the individuals' expectation on positive treatments or interventions, deceptively administrating placebo is considered as a standard approach to ensure that the positive expectation can be established [7]. On the other hand, open-label placebo is being paid close attention to in practice, given its nondeceptive nature [8, 9]. It has been demonstrated that open-label placebo accompanied with positive verbal suggestion and/or a context of supportive patient-practitioner relationship alters pain perception in patients with irritable bowel syndrome (IBS) or chronic low back pain [10, 11]. In addition, once individuals have experienced the pain reduction after receiving a placebo, the disclosure of the placebo would not lead to a failure of the placebo effect [12, 13].

Why placebo treatment is still effective even when the people have known that the treatment is inert? One plausible explanation is that verbal suggestion, supportive patient-practitioner relationship, and conditioning procedure induce additional expectation on the efficacy of the placebo, therefore mediating the effect [2, 4, 14, 15]. However, the extent to which expectation influences the nondeceptive placebo effect is still unclear. If one's expectation underlies the efficacy of placebos, one would expect a reduction or even an elimination of the placebo effect after revealing the nature of the placebo without increased expectation intervention. In other words, an increased expectation of open-label placebo may contribute to the placebo effect, while a decreased expectation may further reduce the effect.

To test this hypothesis, in the present study, different expectation interventions (i.e., no expectation, increasing expectation, and decreasing expectation) were administrated accompanied with the disclosure of the placebo after the participants have experienced the placebo effect. In addition, given that an administration of placebos may not be only lead to placebo effect but also result in nocebo effect [16, 17], a negative response to the treatment [4, 17, 18], the examinations on how expectation interventions influence the nocebo effect were also included to provide insights into the avoidance of the nocebo effect via expectation modulation.

## 2. Methods

### 2.1. Participants

A total of 76 healthy (mean age = 20.89 ± 1.34 years; ranging from 18 to 24 years) volunteers were recruited from the Southwest University, China. Only right-handed female volunteers were recruited in the present study to rule out the possible confounding factors of handedness and gender [19, 20]. None reported with cardiovascular or neurological diseases, family or personal history of psychiatric disorders, acute or chronic pain, color blindness, current use of any medication, or contraindications of electrical stimulation. To avoid confounding effects on pain perception, they were further instructed not to consume products containing caffeine, nicotine, or alcohol 24 h before the experiment [21, 22]. Four participants who were unable to discriminate the distinct levels of electrical pain stimuli used in the conditioning phase were excluded from further investigation. Upon arrival, the Chinese versions of the State-Trait Anxiety Inventory (STAI) [23] were adopted to assess the anxiety state (STAI-S) and anxiety trait (STAI-T), respectively, in all participants. All participants gave their written consents and were informed of their rights to discontinue participation at any time. They were informed that the study aimed at examining the effect of *subliminal electric stimulus equipment* for pain modulation. The experiment procedure was approved by the Ethics Committee of the Southwest University and carried out in accordance with the approved guidelines. This trial is registered with ChiCTR1800014737.

### 2.2. Pain Induction

The painful stimuli were delivered to the inner side of the left forearm through three stainless steel concentric bipolar needle electrodes connected to a constant current stimulator (model DS7A, Digitimer Ltd, Hertfordshire, UK). Each electrode consisted of a needle cathode (length: 0.1 mm, diameter: 0.2 mm) surrounded by a cylindrical anode (diameter: 1.4 mm). All electrodes were located according to an equilateral triangle shape on the inner side of the left forearm, which has been proved to preferentially activate Aδ nociceptive fibers in the superficial skin layers [24–27]. The method of limits (an ascending series of stimuli in steps of 0.1 mA were delivered starting from subtactile threshold until pain sensation was induced) was used to identify the stimulus intensity that would elicit individual pain experience [28]. Each stimulus (mean intensity = 1.21 ± 0.50 mA across all participants) consisted of several succeeding constant current, square wave pulses (2 pulses for low pain, 10 pulses for moderate pain, and 20 pulses for high pain, resp.), with 50 Hz frequency. Before the formal experiment, participants were familiarized with a series of electrical pain stimuli.

### 2.3. Procedures

The experiment consisted of two phases: a conditioning phase and a test phase, with a 10 min break set between the two phases. During the whole procedure, participants wore a sham subliminal electric stimulus equipment on the middle finger of their left hand.

**Figure 1:**
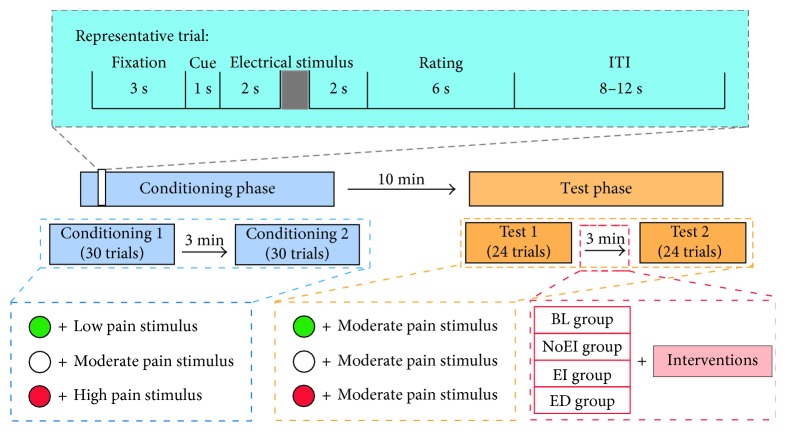
Experimental design. The experiment consisted of two phases: conditioning phase and test phase, separated by a 10 min break. In the conditioning phase, each trial started with a 3 s fixation. Sequentially, a 1 s visual cue was displayed. All participants were told that the visual cue in a certain color was associated with a certain effect (hyperalgesic effect, no effect, or analgesic effect) caused by the equipment. Two seconds after the disappearance of the visual cue, an electrical stimulus was delivered to the participants. Following by a 2 s gap, participants were asked to rate the perceived pain intensity within 6 s. The interval between trials varied from 8 to 12 s. Two sessions of 30 trials were included in the conditioning phase, separately by 3 min blank. In the test phase, the procedure was identical to that in the conditioning phase, except that (1) each session consisted of 24 trials and (2) all visual cues were associated with a moderate pain stimulus. After Test 1, different interventions were given to the four groups.

Participants were sitting approximately 60 cm from a 19-inch monitor (display resolution: 1440 × 900 pixels). As shown in Figure 1, in the conditioning phase, each trial started with a 3 s white fixation cross centered on the screen with black background. Sequentially, a solid circle (diameter: 2 cm), which was red, white, or green in color, was presented on the screen for 1 s. All participants were told that the visual cue in a certain color was associated with a certain effect (hyperalgesic effect, no effect, or analgesic effect) caused by the equipment. To rule out possible confounding effect related to color itself, half of the participants for each group were informed that the green cue was associated with an analgesic effect of the equipment with low-frequency current, the red cue was linked to a hyperalgesic effect of the equipment with high-frequency current, and white cue suggested a deactivation of the equipment. The other half were told the associations between the green cue and a hyperalgesic effect and between the red cue and an analgesic effect. Two seconds after the visual cue disappeared from the screen, a pain stimulus was delivered to the left forearm at low, moderate, or high level to ensure the analgesic and the hyperalgesic effects were established accordingly. Participants were required to verbally rate the perceived pain intensity 2 s later on a 11-point numeric rating scale (0 = no pain at all; 10 = unbearable pain) within 6 s. The interval between trials varied from 8 s to 12 s. This phase consisted of two sessions, Conditioning 1 and Conditioning 2, separated by a 3 min interval. There were 30 trials for each session, with 10 trials for each association between the visual cue and pain stimulus. The sequence of visual cues (red, white, or green) paired with different pain levels (defined as low/moderate/high pain cues, resp.) were counterbalanced across all participants.

For the test phase, the procedure was identical to that in the conditioning phase, except that the intensity of pain stimulus was set at the moderate level (i.e., the intensity associated with the white cue in the conditioning phase) in accordance with a previously described paradigm [29]. To reduce the possible effect of habituation/sensory adaption on placebo/nocebo responses [30, 31], two sessions of 24 trials for each (rather than 30 trials) were included in the test phase, with eight trials for each color of visual cues.

After Test 1, each participant was randomized into one of the four different experimental groups: baseline (BL) group (*N* = 18), no expectation intervention (NoEI) group (*N* = 18), expectation increasing (EI) group (*N* = 18), and expectation decreasing (ED) group (*N* = 18) to receive different interventions as follows:

1. Participants in the BL group were given no intervention.
2. Participants in the NoEI group were told that the subliminal electric stimulus equipment had been closed in Test 1, and the change of pain ratings reflected their placebo and nocebo responses. In the following Test 2, the subliminal electric stimulation equipment would still be closed.
3. Participants in the EI group were told the same instruction as those in the NoEI group, except that they were further told that previous studies indicate that the nondeceptive placebo/nocebo can also change the pain perception.
4. Participants in the ED group were told the same instruction as those in the NoEI group, except that they were further told that previous studies indicated that the placebo/nocebo responses would vanish after the placebo/nocebo has been disclosed.

### 2.4. Statistical Analysis

To compare the characteristics of participants in different experimental groups, we performed one-way analysis of variance (ANOVA) on (1) age, (2) anxiety state (STAI-S), and (3) anxiety trait (STAI-T) using “Group” (BL, NoEI, EI, and ED) as a between-subject factor.

To exclude the difference of pain ratings among groups in the conditioning phase, a two-way repeated measures ANOVA was conducted on the pain ratings, using “Cue type” (low, moderate, and high pain cues) as a within-subject factor and “Group” (BL, NoEI, EI, and ED) as a between-subject factor. To correct the violation of the assumption of sphericity, either the Huynh–Feldt correction (when epsilon > 0.75) or Greenhouse–Geisse correction was applied (when epsilon < 0.75) [32]. Multiple comparisons were adjusted by using the Bonferroni correction, when necessary (the same hereinafter).

To assess whether placebo and nocebo effects were induced successfully, a two-way repeated measures ANOVA was conducted on the pain ratings for Test 1, using “Cue type” (low, moderate, and high pain cues) as a within-subject factor and “Group” (BL, NoEI, EI, and ED) as a between-subject factor.

The decrease of perceived pain intensity, an index of the placebo effect, was obtained by subtracting the ratings paired with low pain cue from those paired with moderate pain cue; the increase of perceived pain intensity, an index of the nocebo effect, was obtained by subtracting the ratings paired with moderate pain cue from those paired with high pain cue. To assess the influence of different interventions on placebo and nocebo effects, we performed two-way repeated measures ANOVA on the placebo effect and nocebo effect, respectively, using “Test” (Test 1 and Test 2) as a within-subject factor and “Group” as a between-subject factor.

In addition, paired-sample *t* tests were performed across groups in Test 1 and Test 2, respectively, to verify the difference between the placebo effect and the nocebo effect.

## 3. Results

### 3.1. Participant Characteristics

Participant characteristics for each experimental group are summarized in Table 1. Results of one-way ANOVA indicated that participant characteristics were not significantly different across groups (age: *F*(3, 68) = 1.96, *P*=0.13, *η*_*p*_^2^ = 0.08; STAI-S: *F*(3, 68) = 0.64, *P*=0.59, *η*_*p*_^2^ = 0.03; STAI-T: *F*(3, 68) = 0.03, *P*=0.99, *η*_*p*_^2^ = 0.001), thus avoiding possible bias due to individual differences when assessing the placebo and nocebo effects.

### 3.2. No Difference of Pain Ratings in the Conditioning Phase

Two-way repeated measures ANOVA revealed that pain ratings in the conditioning phase were significantly modulated by the main effect of “Cue type” (*F*(1.65, 112.02) = 1171.98, *P* < 0.001, *η*_*p*_^2^ = 0.95) but not by the main effect of “Group” (*F*(3, 68) = 0.23, *P*=0.88, *η*_*p*_^2^ = 0.01), and the interaction of the two factors (*F*(6, 136) = 0.68, *P*=0.66, *η*_*p*_^2^ = 0.006) (Table 2). This observation indicated that there was no difference in pain ratings among groups in the conditioning phase.

### 3.3. The Induction of Placebo and Nocebo Effects

To ensure successful inductions of placebo and nocebo effects, a two-way repeated measures ANOVA was conducted on the pain ratings for Test 1. A significant main effect of “Cue type” was found (*F*(1.13, 76.76) = 126.23, *P* < 0.001, *η*_*p*_^2^ = 0.65) with nonsignificant interaction between “Cue type” and “Group” (*F*(6, 136) = 0.15, *P*=0.99, *η*_*p*_^2^ = 0.006). As shown in Table 3, participants reported highest pain scores when the stimuli were presented with high pain cues and lowest pain scores when the stimuli were presented with low pain cues (all *P* < 0.001), indicating the placebo and nocebo effects were induced successfully. This pattern was consistent across groups, as no significant main effect of “Group” was found (*F*(3, 68) = 2.11, *P*=0.11, *η*_*p*_^2^ = 0.09).

### 3.4. The Influence of Expectation on Nondeceptive Placebo and Nocebo Effects

**Figure 2:**
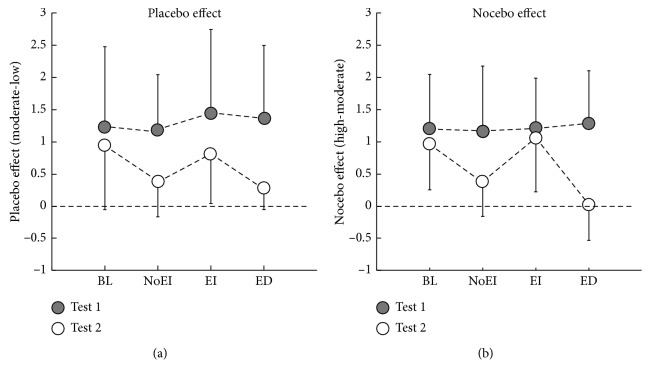
Placebo effect (a) and nocebo effect (b) in Test 1 and Test 2. Error bars indicate one standard deviation, and data from participants in Test 1 and Test 2 are marked in solid and hollow circles, respectively.

A two-way repeated measures ANOVA on the placebo effect showed that a significant main effect of “Test” (*F*(1, 68) = 46.74, *P* < 0.001, *η*_*p*_^2^ = 0.41) and a significant interaction between “Test” and “Group” (*F*(3, 68) = 3.04, *P*=0.04, *η*_*p*_^2^ = 0.12). As can be seen in Figure 2 and Table 4, post hoc analyses showed that participants who underwent no intervention (i.e., BL group) showed comparable placebo effect in Test 1 and Test 2 (*P*=0.22). Participants in the NoEI, EI, and ED groups, on the other hand, showed smaller placebo effect (but still significant when compared with “0” to demonstrate the existence of the placebo effect; all *T*(17) > 2.79 and all *P* < 0.05) in Test 2 when compared with Test 1 (all *P* < 0.003). More importantly, NoEI and ED interventions but not EI interventions were more likely to reduce the placebo effect established in Test 1 when compared to the baseline (both *P* < 0.05). No other significant effects were found.

Similar to the results of the placebo effect, the analysis of the nocebo effect showed a significant main effect of “Test” (*F*(1, 68) = 40.37, *P* < 0.001, *η*_*p*_^2^ = 0.37), along with a significant interaction between “Test” and “Group” (*F*(3, 68) = 7.93, *P* < 0.001, *η*_*p*_^2^ = 0.26). As can be seen in Figure 2 and Table 4, post hoc pairwise comparisons showed that the nocebo effect was smaller for the NoEI group (but still significant when compared with “0” to demonstrate the existence of the nocebo effect, *T*(17) = 2.96, *P*=0.009) and disappeared for the ED group (not significant when compared with “0” to show the disappearance of the nocebo effect, *T*(17) = −0.18, *P*=0.86) in Test 2 in comparison to Test 1 (all *P* < 0.001). Furthermore, the nocebo effect was less affected in the BL and EI groups than in the NoEI and ED groups in Test 2 (all *P* < 0.05, except the *P* value for the comparison between the BL and NoEI groups was 0.06). No other significant effects were found.

### 3.5. The Comparison between Placebo and Nocebo Effects

There were no significant difference between the placebo effect and nocebo effect for each group in Test 1 (BL group: *T*(17) = 0.02, *P*=0.99; NoEI group: *T*(17) = 0.22, *P*=0.83; EI group: *T*(17) = 1.12, *P*=0.28; ED group: *T*(17) = 0.44, *P*=0.67) and in Test 2 (BL group: *T*(17) = −0.07, *P*=0.95; NoEI group: *T*(17) = −0.35, *P*=0.73; EI group: *T*(17) = −1.94, *P*=0.07; ED group: *T*(17) = 1.53, *P*=0.14).

## 4. Discussion

The aim of the present study was to investigate the extent to which expectation affects nondeceptive placebo and nocebo effects. To this end, all participants first underwent a conditioning phase, where after associations between pain stimuli and visual cues were established, as reflected by the placebo and nocebo effects in Test 1. Sequentially, participants were assigned to one of the four experimental groups (i.e., BL, NoEI, EI, and ED) to receive no intervention or different verbal interventions that modulated their expectation of the upcoming pain stimuli in Test 2.

Three main findings emerged in the present study. First, when revealing that the treatment in Test 1 was a placebo, the placebo effect persisted only in the group with increased expectation on the placebo, when compared with the baseline. In contrast, the placebo effect was significantly reduced in groups with decreased or no expectation in compared to that in the BL group. Second, results on the nocebo effect yielded to similar pattern, where the disclosure of the nocebo has neither hindered nor reduced the nocebo effect if expectation increasing intervention was introduced, but it reduced when no expectation was administered and disappeared when decreasing expectation intervention was administered. Third, no difference on effect size between nondeceptive placebo and nocebo effects was found for all groups.

One major finding of the present study is that nondeceptive placebo effect only persisted when individuals were administered with expectation increasing intervention. This observation is partly inconsistent with previous studies showing that nondeceptive placebo treatments could lower participants' disease symptoms and pain perception [10–12], as reduced nondeceptive placebo effects were found in groups with decreased and no expectation intervention when compared with the baseline. It is possible because the claim on effectiveness of nondeceptive placebo is usually based on the manipulations that only positive verbal suggestion was given to participants as open-label placebo (e.g., Carvalho et al.'s study) or the disclosure of placebo just happens after participants have experienced the analgesic effect (e.g., Schafer et al.'s study), which contributes to the placebo effect [33–35]. In other words, different from the present study, the previous studies did not compare situations with increased, decreased, and/or no expectation of placebo effect. From this perspective, our finding extends our current understanding of nondeceptive placebo effect by emphasizing the important role of expectation in the process.

Indeed, it has been proved that expectation is one of the main underlying psychological mechanisms of the placebo effect [3, 4, 36–39]. To evaluate the relationship between expectation and nondeceptive placebo effect, we altered participants' expectation on the effectiveness of placebo by administrating different verbal suggestions. Verbal suggestion is widely used as an approach of expectation modulation. For example, healthy individuals' pain tolerance is increased after receiving suggestion of analgesic placebo given verbally [4]. Verbal suggestion can also change patients' expectations and mediate placebo effects in many diseases [1], such as Parkinson's disease [40], and clinical pain [41, 42].

The modulation of expectation can also affect nondeceptive nocebo effect. For our sample, the disclosure of the nocebo eliminated the established nocebo effect for those receiving decreased intervention and reduced the nondeceptive nocebo effect for those with no expectation intervention but not for those with expectation increasing intervention. It is possible because increased expectation of the upcoming treatments not only leads to the placebo effect but also leads to perceived side effects [4]. In other words, the manipulation on expectation increased group made those participants to believe that they would experience similar placebo and nocebo effects as in deceptive condition (i.e., Test 1). This finding is in good agreement with a recent study suggesting that individuals with higher-priced treatment (usually leads to higher expectation) tend to exhibit stronger nocebo effect [43]. In contrast, for those participants with no or decreased expectation on the nocebo, the disclosure of nocebo may arouse their suspicions of the treatment, thus reducing participants' expectation of nocebo. In brief, compared with nondeceptive placebo effect, nondeceptive nocebo effect might be more susceptible to verbal suggestion.

Deceptive placebo and nocebo are widely administrated in double-blinded experimental settings to evaluate the effectiveness of the target manipulation (e.g., administration of inert substance or sham treatment) without conscious deception [7, 44]. Typically, individuals are induced to believe that the inert substance or sham treatment is effective [44, 45]. Although such a placebo has been widely used in clinical practice [8, 9], giving a placebo intervention deceptively as a treatment is totally unacceptable in some cases [44, 46]. From this perspective, open-label placebos should be used when individuals have positive expectation of the treatment [8, 9, 47]. However, as the efficacy of the placebo may decrease after disclosure, how to maintain or improve the effectiveness of placebos without deception in various clinical situations needs to be further studied.

In addition, side effects caused by nondeceptive placebo and nocebo should be taken into consideration seriously in clinical practice. As previous findings suggested, individuals with increased expectation of the treatment may also show increased nocebo effect [16, 43, 48]. Therefore, when disclosing of pharmacological properties of the drug and possible side effect in the informed consent, there is a risk that it may cause patient's suspicion of the drug effectiveness, resulting in negative response to the treatment [17, 49, 50]. To avoid it, sufficient and rational explanation of the potential side effects is needed.

## 5. Conclusion

The present study provides experimental evidence showing that deception is not necessary to achieve a placebo response [9]. Open-label placebo with positive verbal intervention (i.e., increased expectation) can also alter one's pain perception. In addition, to our knowledge, it is the first study to demonstrate that the modulation of expectation can also affect nondeceptive nocebo effect, which provides an alternative way to avoid side effects of placebo treatment or medical therapy. Overall, our study demonstrated that expectation plays a vital role in nondeceptive placebo and nocebo effects, which calls for future studies focusing on translating the present experimental findings into clinical practice settings.

## Figures and Tables

**Table 1:** Characteristics of participants in each experimental group.

Group	*N*	Age	STAI-S	STAI-T
BL	18	21.06 ± 1.83	39.72 ± 9.43	41.72 ± 5.80
NoEI	18	21.00 ± 1.45	37.72 ± 8.01	42.33 ± 8.04
EI	18	21.50 ± 1.62	36.28 ± 3.98	42.17 ± 6.34
ED	18	21.28 ± 1.60	37.83 ± 7.46	42.28 ± 6.73

Data are expressed as mean ± standard deviation. *N*: number of participants; STAI-S: state subscale of State-Trait Anxiety Inventory; STAI-T: trait subscale of State-Trait Anxiety Inventory.

**Table 2:** Pain ratings of each experimental group in the conditioning phase.

Group	Pain ratings in conditioning phase		
	Low pain cue	Moderate pain cue	High pain cue
BL	1.18 ± 0.66	4.38 ± 1.15	7.22 ± 1.27
NoEI	1.10 ± 0.77	3.83 ± 0.93	7.26 ± 1.23
EI	1.07 ± 0.55	4.07 ± 0.93	7.23 ± 1.06
ED	1.17 ± 0.51	4.16 ± 0.97	7.05 ± 1.11

Data are expressed as mean ± standard deviation.

**Table 3:** Pain ratings of each experimental group in Test 1.

Group	Pain ratings in Test 1		
	Low pain cue	Moderate pain cue	High pain cue
BL	4.43 ± 1.59	5.65 ± 1.36	6.86 ± 1.62
NoEI	3.43 ± 1.47	4.62 ± 1.29	5.77 ± 1.77
EI	3.48 ± 1.37	4.93 ± 1.38	6.14 ± 1.74
ED	3.27 ± 1.88	4.63 ± 1.75	5.92 ± 2.16

Data are expressed as mean ± standard deviation.

**Table 4:** Placebo and nocebo effects of each experimental group in the test phase.

Group	Test 1		Test 2	
	Placebo effect	Nocebo effect	Placebo effect	Nocebo effect
BL	1.22 ± 1.25	1.22 ± 0.83	0.96 ± 1.02	0.98 ± 0.73
NoEI	1.18 ± 0.85	1.15 ± 1.02	0.33 ± 0.50	0.38 ± 0.54
EI	1.45 ± 1.29	1.20 ± 0.78	0.81 ± 0.77	1.08 ± 0.86
ED	1.36 ± 1.13	1.29 ± 0.81	0.23 ± 0.28	−0.02 ± 0.51

Data are expressed as mean ± standard deviation.
